# Factors Associated with Sleep Disorders among Methadone-Maintained Drug Users in Vietnam

**DOI:** 10.3390/ijerph16224315

**Published:** 2019-11-06

**Authors:** Tuan Anh Le, Anh Duc Dang, An Ha Thi Tran, Long Hoang Nguyen, Trang Huyen Thi Nguyen, Hai Thanh Phan, Carl A. Latkin, Bach Xuan Tran, Cyrus S.H. Ho, Roger C.M. Ho

**Affiliations:** 1 National Institute of Hygiene and Epidemiology, Hanoi 100000, Vietnam; lat@nihe.org.vn (T.A.L.); dda@nihe.org.vn (A.D.D.); 2 Institute for Preventive Medicine and Public Health, Hanoi Medical University, Hanoi 100000, Vietnam; bach.ipmph@gmail.com; 3 National Institute of Mental Health, Bach Mai Hospital, Hanoi 100000, Vietnam; antranthiha@bachmai.edu.vn; 4 Center of Excellence in Behavioral Medicine, Nguyen Tat Thanh University, Ho Chi Minh City 700000, Vietnam; longnh.ph@gmail.com (L.H.N.);; 5 Center of Excellence in Pharmacoeconomics and Management, Nguyen Tat Thanh University, Ho Chi Minh City 700000, Vietnam; trang.coentt@gmail.com; 6 Institute for Global Health Innovations, Duy Tan University, Da Nang 550000, Vietnam; 7 Bloomberg School of Public Health, Johns Hopkins University, Baltimore, MD 21205, USA; carl.latkin@jhu.edu; 8 Department of Psychological Medicine, National University Hospital, Singapore 119074, Singapore; cyrushosh@gmail.com; 9 Department of Psychological Medicine, Yong Loo Lin School of Medicine, National University of Singapore, Singapore 119228, Singapore; 10 Institute for Health Innovation and Technology (iHealthtech), National University of Singapore, Singapore 119077, Singapore

**Keywords:** sleep disturbance, sleep disorder, PSQI, methadone, Vietnam

## Abstract

Sleep quality among heroin-dependent patients receiving methadone maintenance treatment (MMT) is not fully investigated in Vietnam. This study explored the prevalence of poor sleep quality in methadone-maintained patients and associated factors. This cross-sectional included 395 MMT patients at three clinics in Nam Dinh province, Vietnam. The Pittsburgh Sleep Quality Index (PSQI) was employed to measure patients’ sleep quality. Sociodemographic, clinical, behavioral, psychological, and social support characteristics were collected. Multivariate Logistic and Generalized Linear Regression models were applied to identify associated factors. Among 395 patients, 26.6% had poor sleep quality according to the PSQI scale. People having jobs were less likely to have poor sleep quality and lower PSQI scores compared to unemployed patients. Those having spouses had lower PSQI scores than single patients. High depression, anxiety, and stress scores were associated with poor sleep quality and high PSQI scores. A longer duration of MMT increased the likelihood of experiencing poor sleep quality. Patients smoking tobacco daily or concurrently using drugs had lower PSQI scores than those that did not. This study highlights a moderate prevalence of poor sleep quality among Vietnamese MMT patients. Regular evaluation, appropriate psychological management, and social support, as well as the provision of employment opportunities, potentially improve the sleep quality of methadone-maintained patients.

## 1. Introduction

Sleep disturbance is a common global health issue given that more than one-third of adults experience this problem during their lifetime [1,2]—a proportion of which may be genetic [3]. Poor sleep quality increases the risk of physical and psychological impairments, as well as chronic morbidities and injuries [1,4,5]. Heroin-dependent individuals are particularly vulnerable to sleep disorders. Prior research found a higher frequency of abnormal cycling patterns of sleep, central sleep apnea, and higher variance of respiratory rate during sleep—reflecting sleep-disordered breathing—among these people compared to the healthy control group [6,7,8]. Another reason might be because using heroin and other opiates could affect the opioid receptor μ-1 (OPRM1), which decreases adenosine concentration levels—an important neurochemical driving factor for sleep, leading to sleep impairment [9,10]. Furthermore, Wang et al. found that OPRM1 could be directly related to sleep disturbance in patients receiving methadone maintenance treatment (MMT) through several single nucleotide polymorphisms [11]. Otherwise, using heroin and other opiates releases acetylcholine in the brain, inhibiting the rapid eye movement (REM) sleep phase, suppressing the transmission of gamma-aminobutyric acid-ergic (GABAergic) in the dorsal raphe nucleus, and finally resulting in patients’ wakefulness [12,13].

Sleep problems have been documented to elevate the risk of drug use relapse and polysubstance use among heroin-dependent individuals, especially those receiving MMT [14,15,16]. The MMT program is a comprehensive initiative using methadone as an alternative to the opiate on which the patient was dependent before. This program consists of counseling, management, and other medical services that can help opioid-dependent individuals to diminish opioid withdrawal symptoms [17]. Nevertheless, in MMT patients, sleep problems cause methadone detoxification and withdrawal symptoms, reduce addiction treatment adherence, and eventually diminish the addiction treatment effectiveness and the patients’ quality of life [18,19,20,21]. In the literature, the pervasiveness of sleep problems among MMT patients has been well-recognized, with the prevalence varying from 70% to 99% [15,22,23,24]. Another cohort in Taiwan revealed that 19.1% of MMT patients developed sleep problems within two years of treatment [25]. Predictors of sleep problems include the duration of opioid use, psychological problems, substance use and abuse, MMT dosage, and MMT duration [15,22,23,24]. With the high variability of rates of sleep problems and their determinants in MMT patients across settings, assessing this health issue and associated factors are particularly important to develop strategies for optimizing the effectiveness of MMT.

In Vietnam, the MMT program has been implemented since 2008 and has since expanded massively to 63/63 provinces, with 294 MMT clinics and approximately 53,000 patients in September 2017 [26]. The program’s activities mostly rely on foreign aid, which has been rapidly cut in recent years [26]. Therefore, efforts for maximizing the benefits of the MMT program are encouraged, consisting of improving treatment effectiveness and reducing adverse effects such as sleep disturbance. However, empirical evidence about sleep quality and associated factors among people using MMT in Vietnam is lacking. This study was conducted to explore the prevalence of poor sleep quality in methadone-maintained patients in some MMT clinics and determine factors associated with poor sleep quality.

## 2. Materials and Methods

### 2.1. Study Design and Sampling

A cross-sectional study was conducted in the Nam Dinh province from January to September 2018. There were five MMT clinics operated in the province, and three MMT clinics were randomly selected as study settings, including: (1) Giao Thuy district health center, (2) Dai Dong private health facility, and (3) Giao Thuy Center for Social Evils Prevention. These clinics provide MMT services following the official guidelines of the Vietnam Ministry of Health for at least 12 months. Patients were conveniently recruited if they were: (1) aged at least 18 years old; (2) enrolled in an MMT program in selected clinics; (3) accepted to participate and gave verbal informed consents; (4) did not have cognitive impairments (according to physicians’ diagnosis) that might affect patient communication with the interviewers. Among the 400 patients invited, 395 patients accepted to participate (response rate of 98.8%). Because the clinics only had male patients, 100% of our samples were males.

### 2.2. Measure and Instruments

All patients were involved in 20-minute face-to-face interviews with well-trained researchers from the National Institute of Hygiene and Epidemiology and the Nam Dinh Provincial Center for HIV/AIDS Control and Prevention. They were invited to participate when they visited clinics for medication. They were informed briefly about the aim of this study as well as the significance and disadvantages of participation. If they agreed to be enrolled, they were invited to go to a private counseling room in the clinic and asked to give written informed consent. A structured questionnaire was used for data collection. This tool was piloted with 20 MMT patients to ensure the language and logical order of each question. The questionnaire measured the information detailed in the following subsections.

#### 2.2.1. Primary Outcome 

Each participant was administered the Pittsburgh Sleep Quality Index (PSQI)—a 19-item questionnaire to assess sleep quality in seven components, including subjective sleep quality, sleep latency, sleep duration, habitual sleep efficiency, sleep disturbances, use of sleeping medications, and daytime dysfunction during the last month. The questionnaire has been used and validated in various settings for the measurement of sleep quality and pattern among adults with Cronbach’s alpha = 0.77. Each component receives a score from 0 (none during the past month) to 3 (at least three times a week). The final score ranged from 0 to 21. A higher score represents worse sleep quality, and scores >5 indicate poor sleep quality [27].

#### 2.2.2. Covariates

Personal information consisting of age, gender, education, marital status, occupation, and monthly household income was collected. We also obtained self-reported behaviors and characteristics about concurrent drug use (“Have you used any drugs in the last 30 days?”) and the frequency of alcohol use (“How often do you use alcohol in the last 30 days?”) and smoking tobacco (“How often do you smoke tobacco in the last 30 days?”). For clinical characteristics, we extracted medical records to collect data about MMT duration, current MMT dose, and the number of missed doses in the last 30 days. The Depression, Anxiety, and Stress Scale-21 (DASS-21) was used to evaluate depression, anxiety, and stress in the last seven days among patients. This tool has 21 items with seven items per subscales and four levels of response (0—“Did not apply to me at all” to 3—“Applied to me very much, or most of the time”) per item. The total score of each subscale ranges from 0 to 21, which lower score indicates better status [28]. The Vietnamese version of this instrument has been used and validated previously, with Cronbach’s alpha ranging from 0.761 to 0.906 in the adolescent population [29] and from 0.70 to 0.88 among Vietnamese women [30]. We also asked patients to report whether they received any support from health staff/relatives/peers/other people for medication.

### 2.3. Statistical Analysis

Data were analyzed by STATA version 12. Chi-square and Mann Whitney tests were utilized for analyzing the differences between patients with and without poor sleep quality according to various factors. We applied a multivariate logistic regression model for identifying factors associated with poor sleep quality (Yes/No). Moreover, a generalized linear regression model to determine related factors to PSQI score (continuous variable), with the Gaussian family and identity link, was also employed. Forward stepwise selection strategies (with a *p*-value of 0.2) were used to build final reduced models. A *p*-value < 0.05 was considered as statistical significant.

### 2.4. Ethical Approval

Ethics approval was approved and granted by the Institutional Review Board of National Institute of Hygiene and Epidemiology (Code: 726/QDVSDTTU).

## 3. Results

Table 1 depicts that 26.6% had poor sleep quality according to PSQI scale. The mean age of the sample was 25.9 (Standard Deviation (SD) = 7.8) years. Most of the patients had secondary education (60.0%) and had a spouse (68.6%). The difference between patients with and without poor sleep regarding education, age, and marital status was not statistically significant (*p* > 0.05). Meanwhile, those that experience poor sleep had a higher proportion of unemployment (16.2%) compared to those that did not experience poor sleep (5.5%) (*p* < 0.05).

Table 2 shows that most participants had negative results in the HIV test (92.7%). No difference was found between patients with and without poor sleep regarding ever injected drugs, concurrent drug use, alcohol use, smoking tobacco, MMT duration, MMT doses, and MMT missed doses (*p* > 0.05). The depression, anxiety, and stress scores according to the DASS-21 scale of participants who had poor sleep quality were significantly higher than those without poor sleep quality.

In Table 3, the most common sources of support were family members (77.7%), health staff in the MMT clinic (41.3%), and peers (17.0%). Those experiencing poor sleep had a higher proportion of receiving support from neighborhood/friends (7.6%) compared to their counterparts (2.8%) (*p* < 0.05). The majority of the sample received MMT in Public facility (50.6%).

Table 4 presented the associated factors with the sleeping quality of MMT patients. Only variables with p-value less than 0.2 were presented in the table. People who were self-employed, blue collar/farmer, business or had other jobs were less likely to have poor sleep quality and lower PSQI score compared to unemployed patients. Those that had a spouse also had a lower PSQI score than single patients (Coefficient (Coef.) = −0.43; 95% Confidence Interval (CI) = −0.85; −0.01). Patients who smoked tobacco daily (Coef. = −0.59; 95% CI = −1.14; −0.03) or currently used drug (Coef. = −1.22; 95% CI = −2.22; −0.22) had a lower PSQI score than those that did not smoke or used drug, respectively. In addition, depression, anxiety, and stress scores were found to be associated with poor sleep quality, as well as with a high PSQI score. People having a higher duration of MMT (Odds Ratio (OR) = 1.12; 95% CI = 1.001; 1.25) increased the likelihood of reported poor sleep quality.

## 4. Discussion

This study enriches the current literature by informing the prevalence of poor sleep quality and its determinants among patients receiving MMT in Vietnam. In this study, we found a moderate prevalence of poor sleep quality in methadone-maintained patients. Moreover, this study underlined the roles of multiple factors, including psychological problems, employment, social support, and substance use, affecting the levels of sleep quality in this population. These results suggest further implications to improve the effectiveness of the MMT program in Vietnam.

Our study indicated that more than one-fourth of MMT patients (26.6%) suffered from poor sleep quality. This result was lower than other studies in China, Taiwan, and Israel (70% to 99% [15,22,23,24]) and comparable to the opioid-naïve population in Malaysia (30.8%) [31] and a recent study in China (35.2%) [32]. Our finding was similar to the result in Australian and Hong Kong general populations (32% [33] and 39% [34], respectively), but lower than certain occupational populations, such as police officers (51%) [35] or drivers (53.4%) [36]. These disparities might be attributable to different study design characteristics, such as sampling technique (i.e., random sampling or convenient sampling) or clinical features (i.e., inpatients or ambulatory patients). Moreover, several characteristics of our sample would be different such as unemployment rate [37], social support or loneliness [38], anxiety, depression, and stress [39,40], which are potential confounding factors. In this study, we observed a high proportion of patients having spouse/sex partners and receive support from family members (77.7%), which might reduce the risk of poor sleep quality in our patients [21,32,41].

Findings in this study aligned with previous works when showing that psychological problems increased the likelihood of having poor sleep quality in MMT patients [21,41]. It might be due to the hyperactivity of the hypothalamic-pituitary-adrenal (HPA) axis, which was activated when patients experienced depression or stressful events in their lives such as unemployment, leading to hyperarousal, concealing slow-wave sleep (SWS) and causing poor sleep [42,43,44]. Therefore, controlling and managing psychological problems are critical to improving sleep quality of MMT patients. Furthermore, we found that patients having longer MMT duration were at significantly higher risk of having poor sleep quality. This issue can be justified by the fact that long-term utilization of exogenous opiates (e.g., methadone) reduces the endogenous opioid peptides, increasing the latency and delaying the initiation of sleep [41,45,46]. Moreover, literature underlined that methadone use is associated with sleep disorders such as obstructive apnea [47,48]. Notably, our results were inconsistent with previous studies, which found that patients that smoke daily and concurrently using drugs had lower PSQI scores [49]. However, a study in Taiwan found no association between nicotine dependence and poor sleep quality [25]. We supposed that these patients might have a feeling of pleasure when using tobacco and drugs, which could support them to sleep. Further clinical studies should be conducted to confirm the relationships between tobacco and drug use with sleep quality in MMT patients.

This study suggested several implications. First, physicians might apply several approaches such as cognitive-behavioral therapy to improve the sleep quality of patients reporting poor sleep quality. Moreover, they should routinely assess the sleep quality of MMT patients, especially those with psychological problems and having a long duration of MMT, in order to lessen the adverse impacts of sleep problems on patients’ health. Second, patients should be advised about not concentrating on the short-term benefits of drug use and smoking tobacco on good sleep quality but emphasized the long-term harms of substance use on health. Third, there should be more focus on programs that offer vocational training and employment opportunities for unemployed patients, which helps patients to stabilize their lives and reduce anxiety/stress, and as a consequence increase their sleep quality. Finally, MMT clinics should consider organizing training sessions for people who support patients in treatment about how to help patients dealing with sleep problems in systematic and effective manners.

Our strength included the use of validated international measures, namely PSQI and DASS-21, as well as objective data extracted from medical records. It enhances our comparability to studies in other settings. However, several limitations should be noted. First, our findings were drawn from a cross-sectional study, which did not allow us to build the causal conclusions between sleep quality and its determinants. Second, a small sample size recruited conveniently might diminish our statistical power and ability to generalize to other MMT populations in different settings. In addition, we performed multiple comparisons, which might increase the risk of type 1 error. Moreover, several indicators, such as comorbidities or severity of pain, were not collected, which could be potential factors associated with sleep quality. Characteristics of sleep disorder such as abnormal cycling patterns of sleep, central sleep apnea, and variance of respiratory rate should also be fully investigated in further studies. Furthermore, lacking control groups could reduce our ability to measure the impact of MMT on the sleep quality of the drug-use population compared to healthy individuals. Finally, some information was self-reported, which might result in recall bias.

## 5. Conclusions

In conclusion, this study highlights a moderate prevalence of poor sleep quality among Vietnamese MMT patients. Regular evaluation, appropriate psychological management, and social support, as well as employment opportunities provision, have the potential to improve the sleep quality of methadone-maintained patients.

## Figures and Tables

**Table 1:** Demographic characteristics of participants.

Characteristics	Poor Sleep				Total		*p*-Value
	Yes		No				
	*N*	%	*n*	%	*n*	%	
**Total**	105	26.6	290	73.4	395	100.0	
**Education**							
Less than secondary	15	14.3	51	17.6	66	16.7	0.70
Secondary school	66	62.9	171	59.0	237	60.0	
More than secondary	24	22.9	68	23.5	92	23.3	
**Marital status**							
Single	18	17.1	49	16.9	67	17.0	0.79
Having sex partner	11	10.5	22	7.6	33	8.3	
Having spouse	69	65.7	202	69.6	271	68.6	
Divorced/widow	7	6.7	17	5.9	24	6.1	
**Occupation**							
Unemployment	17	16.2	16	5.5	33	8.4	<0.01
Self-employed	30	28.6	109	37.6	139	35.2	
Blue collar/farmer	29	27.6	63	21.7	92	23.3	
Business	11	10.5	17	5.9	28	7.1	
Others	18	17.1	85	29.3	103	26.1	
	**Mean**	**SD**	**Mean**	**SD**	**Mean**	**SD**	
**Age**	26.3	7.6	25.8	7.9	25.9	7.8	0.55

SD: Standard Deviation.

**Table 2:** Clinical and behavioral characteristics of patients.

Characteristics	Poor Sleep				Total		*p*-Value
	Yes		No				
	*n*	%	*n*	%	*n*	%	
**Ever injected drugs**	64	61.0	188	64.8	252	63.8	0.48
**Concurrent drug use**	10	9.5	13	4.5	23	5.8	0.06
**Frequency of alcohol drink in the last 30 days**							
No	57	54.3	127	43.7	184	56.6	0.14
Every few weeks	15	14.3	57	19.7	72	18.2	
Every week	13	12.4	28	9.7	41	10.4	
Daily	20	19.0	78	26.9	98	24.8	
**Frequency of smoking tobacco**							
No	29	27.6	46	15.9	75	19.0	0.07
Every few weeks	5	4.8	15	5.2	20	5.1	
Every week	6	5.7	23	7.9	29	7.3	
Daily	65	61.9	206	71.0	271	68.6	
**Missed any dose in the last 30 days**	9	8.6	27	9.3	36	9.1	0.82
	**Mean**	**SD**	**Mean**	**SD**	**Mean**	**SD**	
**Age at onset of drug use**	26.3	7.6	25.8	7.9	25.9	7.8	0.42
**MMT duration (years)**	3.1	2.2	2.7	2.2	2.8	2.2	0.06
**MMT dose (mg)**	70.3	50.8	68.8	30.6	69.2	37.0	0.34
**MMT missed dose in the last 30 days**	0.1	0.4	0.3	1.8	0.3	0.1	0.80
**DASS**-**21 subscales**							
**Depression**	2.4	4.9	1.2	2.4	1.5	3.1	<0.01
**Anxiety**	4.8	4.5	3.1	3.6	3.6	3.9	<0.01
**Stress**	3.2	4.5	1.7	3.0	2.1	3.5	<0.01

MMT: Methadone Maintenance Treatment; DASS: Depression, Anxiety, and Stress Scale.

**Table 3:** Source of support, MMT model.

Characteristics	Poor Sleep				Total		*p*-Value
	Yes		No				
	*n*	%	*n*	%	*n*	%	
**Source of support**							
None	23	21.9	65	22.4	88	22.3	0.91
Health staff in MMT clinic	38	36.2	125	43.1	163	41.3	0.22
Health staff in commune health center	3	2.9	4	1.4	7	1.8	0.33
Other health workers	0	0.0	1	0.3	1	0.3	0.55
Family members	82	78.1	225	77.6	307	77.7	0.91
Peers	16	15.2	51	17.6	67	17.0	0.58
Neighborhood, friends	8	7.6	8	2.8	16	4.1	0.03
**MMT model**							
Private facility	46	43.8	149	51.4	195	49.4	0.18
Public facility	59	56.2	141	48.6	200	50.6	

**Table 4:** Factors associated with sleeping quality among MMT patients.

Characteristics	Poor Sleep		PSQI Score	
	PR	95% CI	Coef.	95% CI
**Education (versus < Secondary school)**				
Secondary school	1.53	0.91; 2.58		
**Occupation (versus Unemployed)**				
Self-employed	0.24 *	0.10; 0.57	−1.19 *	−2.26; −0.13
Blue collar/farmer	0.41 *	0.17; 0.98	−1.07	−2.17; 0.03
Business	0.46	0.15; 1.40	−1.62 *	−2.80; −0.43
Others	0.15 *	0.06; 0.38	−1.35 *	−2.44; −0.26
**Marital status (versus Single)**				
Having a sex partner	1.90	0.81; 4.43		
Having a spouse			−0.43 *	−0.85; −0.01
**Frequency of alcohol use in the last 30 days (versus None)**				
Daily	0.61	0.34; 1.11		
**Frequency of smoking tobacco in the last 30 days (versus None)**				
Every few weeks			−0.89	−1.86; 0.08
Every week			−0.58	−1.29; 0.12
Daily			−0.59 *	−1.14; −0.03
**Current drug use (Yes versus No)**	0.46	0.17; 1.23	−1.22 *	−2.22; −0.22
**Ever drug injection (Yes versus No)**	1.45	0.87; 2.42	0.34	−0.09; 0.78
**DASS-21 subscales**				
Anxiety score	1.08 *	1.01; 1.16	0.13 *	0.07; 0.20
Depression score			0.10 *	0.03; 0.17
Stress score	1.10 *	1.02; 1.18		
**MMT doses**			0.00	−0.00; 0.01
**Duration of MMT**	1.12 *	1.001; 1.25	0.07	−0.01; 0.15
**Source of support**				
Health staff in MMT clinic (Yes versus No)			0.34	−0.11; 0.79
Other health workers (Yes versus No)			−0.90 *	−1.49; −0.31
Family members (Yes versus No)			0.36	−0.14; 0.85
Neighborhood, friends (Yes versus No)	3.02 *	1.03; 8.81		
MMT model (Public versus Private)			0.27	−0.10; 0.64

PSQI: Pittsburgh Sleep Quality Index; PR: Prevalence Ratio; * *p* < 0.05.
